# Removal Properties of Anionic Dye Eosin by Cetyltrimethylammonium Organo-Clays: The Effect of Counter-Ions and Regeneration Studies

**DOI:** 10.3390/molecules23092364

**Published:** 2018-09-15

**Authors:** Fethi Kooli, Yan Liu, Mostafa Abboudi, Souad Rakass, Hicham Oudghiri Hassani, Sheikh Muhammad Ibrahim, Rawan Al-Faze

**Affiliations:** 1 Community College, Taibah University-Al-Mahd Branch, Al-Mahd 42112, Saudi Arabia; 2 Institute of Chemical and Engineering Sciences, 1 Pesek Road, Jurong Island, Singapore 627833, Singapore; liu_yan@ices.a-star.edu.sg; 3 Department of Chemistry, Taibah University, POBox 30002, Al-Madinah Al-Munawwarah 41147, Saudi Arabia; abboudi14@hotmail.com (M.A.); rakass_souad@yahoo.fr (S.R.); oudghiri_hassani_hicham@yahoo.com (H.O.H.); mdibrahi@gmail.com (S.M.I.); rawan.faze@gmail.com (R.A.-F.); 4 Département de Chimie, Faculté des Sciences Dhar El Mahraz, Université Sidi Mohamed Ben Abdellah, B. P. 1796 (Atlas), Fès 30003, Morocco

**Keywords:** cetyltrimethylammonium cations, organo-clays, counter-ions, exchange reaction, in-situ XRD, anionic dye, eosin dye, removal, regeneration

## Abstract

The organo-clays (OCs) were prepared by a cation exchange reaction between surfactant (cetyltrimethylammonium, C16TMA) from different counterions (Bromide, Chloride, and Hydroxide). The effect of the counterions was investigated on the physico-chemical properties of the prepared organo-clays. The highest uptake of organic cations (1.60 mmol/g) was achieved using cetyl trimethylammonium bromide solution and the lowest value (0.93 mmol/g) was obtained after modification with cetyl trimethylammonium hydroxide solution starting from the same initial ratio of mmol/g of clay greater than 2.40. The arrangement of C16TMA cations within the interlayer space was assumed to be perpendicular with a tilt angle of 32° to the plane of clay sheets instead of being parallel to the clay surface using C16TMAOH solution at the same ratio. Different techniques were used to characterize these materials. The thermal stability of these organ-clays was investigated using an in-situ X-ray diffraction (XRD) technique. The decomposition of the surfactant moiety occurred at temperatures higher than 215 °C and was accompanied with a shrinkage of the basal spacing value to 1.42 nm. These materials were applied in the removal of an acid dye “eosin.” The removed amount of eosin depended on the initial concentrations and the content of surfactants in the organo-clays. The removal of eosin was found to be an endothermic process. The maximum amount of 90 mg/g was achieved. The preheated treatment temperature of two selected OCs did affect the removal properties of eosin. A progressive reduction was observed at temperatures higher than 200 °C. The regeneration of spent OCs was studied and acceptable removal efficiency was maintained after 4 to 6 cycles depending on the used initial concentrations.

## 1. Introduction

Currently, the organoclays (OCs) are attracting a lot of interest either in the academic field or in their wide applications in different industrial areas due to their availability on a large scale such as paints, rheological control agents, grease, personal care, oil well drilling, polymer nanocomposites, etc. [1,2,3]. One of the major applications of these materials is an environmental application and, especially in the polluted water treatment, they are used as an adsorbent or removal agent of neutral organic compounds [4,5] and colorant dyes [6]. The hydrophilic character of clay minerals was modified by surfactants with different chemical structures. The surface nature is changed from hydrophilic to organophilic and these materials have an affinity to non-polar molecules and others [7]. The OCs are generally prepared from clay minerals that belong to 2:1 type (smectite group) due to their high swelling capacities. This property was related to the interlayer small cations and their easy hydration [8]. When clay is in contact with water, these charge-compensating cations can be exchanged or replaced by others present in the bulk of the suspension [5]. The negative surface charge of clay minerals was created by the isomorphous substitution of Al^3+^ for Si^4+^ in the tetrahedral layer and Mg^2+^ for Al^3+^ in the octahedral layer [5]. The properties of clay minerals that makes it ideal for the preparation of OCs are the high surface area and the surface charge. The quaternary alkyl ammonium cations (QACs) are the preferred surface modifiers due to their low cost and easier processing [9]. QACs cations are composed of a nitrogen head group and four exchangeable positions that can be occupied by organic functional groups with different structures and sizes [10].

OCs are usually divided into two groups depending on the length of QACS and the sorption mechanism. The first group is the adsorptive OCs that consist of short chain quaternary ammonium ion (R < 12) such as TMA (tetramethyl ammonium) and TBA (tetrabenzyl ammonium). The second type of OCs is organophilic clays, which consists of a long chain quaternary ammonium ion (R > 12) such as HDTMA (hexadecyl trimethyl ammonium) and ODTMA (octadecyl trimethyl ammonium [11]. The properties of the OCs can be engineered and depended mainly on the choice of the clay minerals, the cation exchange capacity (CEC), and the structure of the surfactants. It has been found that the structure and chemical characteristics of the clay mineral have a strong effect on the cation exchange that is involved in the synthesis of the OCs [12]. Usually the cation exchange reaction starts at the edges of clay particles and then extends to the center. Kinetic studies show that the reaction between the quaternary ammonium salts and the clay mineral improves with the increased temperature [13]. The literature survey indicated that the frequently used alkyl ammonium cations is the cetyl trimethylammonium or hexadecyl trimethyl ammonium (C16TMA) with an alkyl chain of 16 carbons and bromide as an anion. The resulting OCs were prepared from C16TMABr solutions at different concentrations and conditions [14].

Few studies have reported the use of cetyl trimethyl ammonium chloride [15,16,17]. The counterions affected the critical micelle concentration (cmc) of the surfactant solution. The C16TMABr solution has a cmc value of 0.94 mM lower than the cmc of C16TMACl (1.4 mM) in deionized water due to the smaller counterion Br^−^ compared to the Cl^-^ counter ion [18]. These anions also affected the micelle sphere to rod transition in solution [19], the interaction between the cationic surfactants, and the anionic dyes. [20]. The higher pH values of the surfactant solution have affected the exfoliation properties of a particular acid-activated clay mineral. [21]. The use of C16TMAOH solution was undertaken for further studies and interesting data were obtained using acid-activated layered silicates such as clay mineral and protonated magadiite [22,23]. The content of the surfactants cations was improved in the resulting organo-acid activated clays and organo-magadiites [22,23].

Our interest is to use these OCs to remove acidic dyes. The acidic dyes exhibited a negative charge, which make it difficult to be adsorbed on the negatively charged surfaces of clay minerals and other silicate materials due to the electrostatic repulsion [24,25]. Similar observations were reported in case of some agricultural waste materials and the surface modification was able to enhance the affinity of these materials towards anionic dyes. The cationic surfactant (C16TMA^+^) was proposed as the modifying agent for the previously mentioned purpose [26,27].

In this study, the effect of the counter-ions on the intercalation of C16TMA cations was investigated by selecting a clay mineral with higher cation exchange capacity. The synthesis and characterization of the resulting OCs were undertaken by different techniques. The thermal stability of selected OCs based on their organic contents was investigated in-situ X-ray diffraction (means atreal temperatures during the collection of the patterns without cooling down the samples). This part will give an insight about the structural changes in the interlayer spacing and how they might affect the removal properties of an anionic dye “eosin.” The eosin was chosen as a model pollutant because of its variety of usage. It is mainly used in silk, wool, modified acrylic, polyamide, propylene fibers, in pharmaceutical industry, etc. [28,29]. The eosin molecule carries aromatic and sulphonic groups that make these dyes harmful to human and other microorganisms [30]. Different parameters were investigated as well as their effect on the eosin removal such as removal temperature, initial concentrations, and the preheated temperatures of selected OCs.

## 2. Experimental Part

### 2.1. Materials

**Figure 1:**
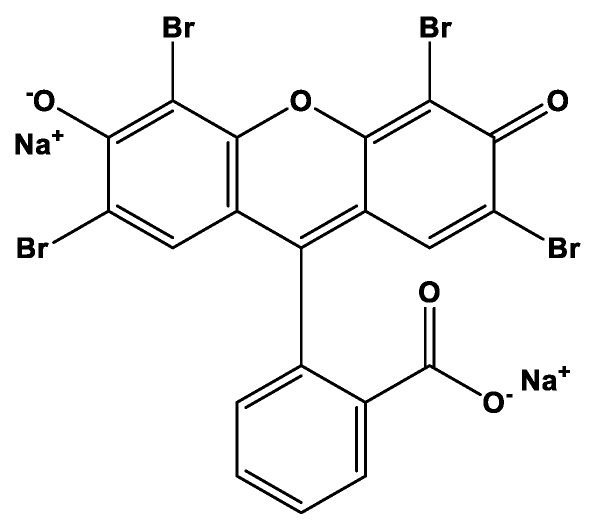
Chemical structure of the eosin Y dye.

A polymer grade montmorillonite (PG) used in this study was obtained from Nanocorcompany (Hoffman Estates, IL, USA) and used without any treatment. Its cation exchange capacity (CEC) value about 145 meq/g as mentioned by the supplier. Cetyl trimethylammonium hydroxide solution (C16TMAOH) was purchased from TCI, Singapore, C16TMABr, C16TMACl solid salts and eosin dye were obtained from Acros Organics (Loughborough, UK) and were used without any purification. Figure 1 presents the chemical structure of the used eosin dye.

### 2.2. Synthesis of OCs

OCs were prepared by a cation exchange reaction [31]. The QACs (known amounts) were dissolved into 25 mL of deionized water.Then 1.0 g of PG clay was poured to the QAC solution and stirred overnight at room temperature. The solution was filtrated and the resulting OCs were washed several times with deionized water and then dried at room temperature overnight.

For comparison purposes, the same molar amounts of C16TMA cations from different sources were used and the corresponding amount of the salt was dissolved in 25 mL of deionized water. Whenever necessary to completely dissolve the surfactants, the solution was gently heated at 50 °C. The OCs were prepared using the same procedure described above. The resultant modified PG clay were denoted as C16XPG-Y where X is the counterion of the used surfactant solution and Y is the initial loading concentration of surfactant in mmol/g (basis).

### 2.3. Effect of Washing Solution

Selected organo-clay (C16BrPG-2.40) was prepared from the C16TMABr solution and, using a loading concentration of 2.40 mmol dissolved into 25 mL after the exchange reaction, the solid was collected by filtration and washed by 100 mL mixture of ethanol and water at different compositions. Then it was dried at room temperature.

### 2.4. Eosin Removal Experiment

The removal of eosin was carried out using batch mode experiments, as reported previously [32]. A fixed amount of OC (0.1 g) was added to 10 mL of known concentrations in a series of 12 mL closed tubes. The used solutions varied from 25 to 1200 mg/L and were prepared by dilution from a stock solution (of 1500 mg/L). The tubes were shaken at 130 rpm at 25 °C for overnight. After centrifugation, the amounts of the un-removed dye in the supernatant solutions were analyzed for its dye concentration using a double beam UV-vis spectrophotometer. All measurements were done at the maximum absorbance at the wavelength of 610 nm.

The removal experiments were conducted by varying initial eosin concentration, temperature of the removal process, and the preheat treatment of selected OCs prior to the eosin removal.

### 2.5. Regeneration/Removal Cycles

Fresh OC samples were mixed with eosin solution (C_i_ = 200 mg/L). After the removal process, the sample was separated by centrifugation. The spent material was treated in an aqueous solution of oxone and cobalt nitrate [33]. The oxone was used as an oxidant and Co^2+^ ions were utilized as a catalyst after separation. The treated solid was added to a fresh dye solution for removal in the succeeding cycle. The same procedure was undertaken for the cycle.

### 2.6. Characterization Methods

The d_001_ spacing of the OCs were estimated from the first *001* reflections in the powder X-ray diffraction patterns and recorded in a Bruker Advance 8 equipped with a Copper anode (Cu Kα = 1.5418 Å). A EURO EA elemental analyser was employed to determine the amount of C16TMA cations taken up by the different samples. Two parallel runs for each sample were performed.

The thermogravimetric analysis was carried out on a TGA/DTA system (TA instrument, model 2690) form RT to 900 °C at a heating rate of 10 °C/min under the air atmosphere. The nitrogen isotherms adsorption was performed on a Micromeritcs ASAP 2040 instrument. The samples were treated at 100 °C under vacuum prior to the measurement. The BET equation was used to estimate the specific surface areas. The pore volume was deduced at a relative pressure (P/P_o_) value of 0.95. The solid ^13^C CP NMR technique was performed to investigate the conformation of the intercalated C16TMA cations. The detail analysis was reported in our previous work [21].

The powder in-situ X-ray diffraction runs were performed using a HTK 16 high temperature chamber (Anton Paar) mounted on a Bruker AXS, D8 Advance diffractometer. The temperature varied in the range of 25 °C to 420 °C. UV-VIS spectrophotometer from Varian (carry 100) was used to estimate the absorbance at maximum wavelength (λ_max_ = 610 nm) in the supernatant and computed from the calibration curves.

## 3. Results and Discussion

### 3.1. Elemental Analysis

The C, H, N elemental analysis was employed to estimate the up take (real) amount of C16TMA cations in the OCs. The data are presented in Table 1. For an initial loading concentration of 2.40 mmol (corresponded to initial CEC/mmol of C16TMA ratio of 1.65), the highest content (39.23%) was obtained when C16TMABr solution was used, and the lowest one (19.01%) was achieved starting from C16TMAOH solution. Using the chloride solution, an intermediate value of 30.77% was attained.

The up take amount of C16TMA cations was higher than the CEC from Br solution. Then it dropped to 86% and 64% for Cl and OH solutions. Attempts have been carried out to improve the content of C16TMA^+^ cations from hydroxide solution by increasing the initial loading concentrations higher than 2.40 mmol. However, the up take amount was unchanged. Similar data were obtained for another type of clay mineral with low cation exchange capacity (CEC) [34].

**Figure 2:**
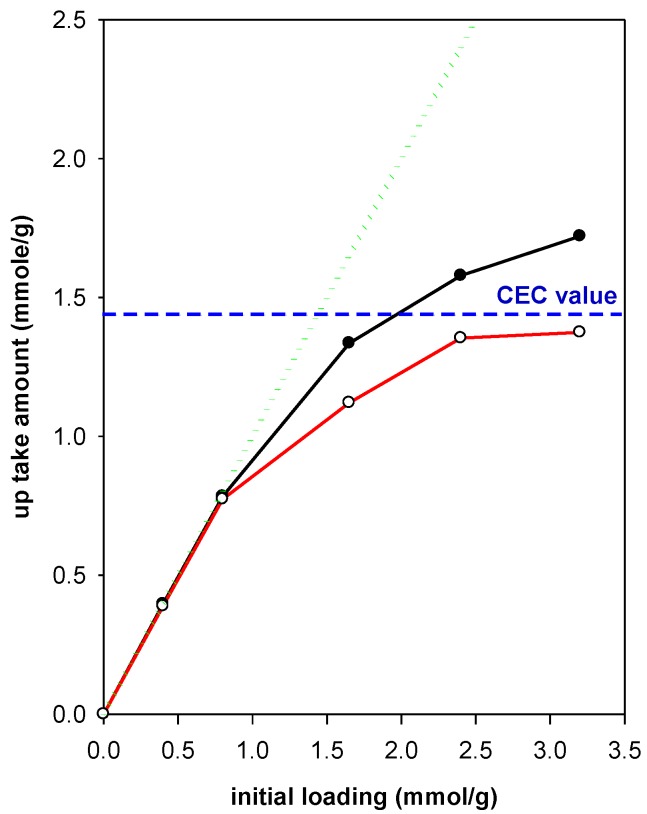
The uptake amount of C16TMA cations by the PG clay as a function of initial loading concentrations using C16TMABr solution.

Using a C16TMABr solution, the up take amount was tuned by changing the initial loading concentrations below 2.40 mmol. The data are presented in Figure 2. All the C16TMA cations initially loaded were taken up by the PG clay mineral for the initial ratios CEC to mmol close to 1 (Y values less than 1.65). At higher ratios, the experimental up take values were higher than the CEC values but are still lower than the theoretical values (Figure 1).

These data indicated that the mechanism of C16TMA uptake using bromide solution was different than the hydroxide one. The later one was mainly based on a cation exchange reaction. However, using Br solution, an additional mechanism happened and could be related to the hydrophobic interaction between the surfactant molecules [35].

This mechanism was modified by using the same initial loading concentration (sample C16BrPG-2.40) and by washing the resulting OC by different mixtures of ethanol and water. The C, H, N analysis are summarized in Table 2 and indicated that the content of the surfactants in the obtained OCs decreased when it was washed with a mixture of ethanol and water even at a low percentage of ethanol of 25%. Similar observations were noted when using a mixture of ethanol and water as a reaction medium instead of pure deionized water [21,36,37].

### 3.2. Powder XRD Data

**Figure 3:**
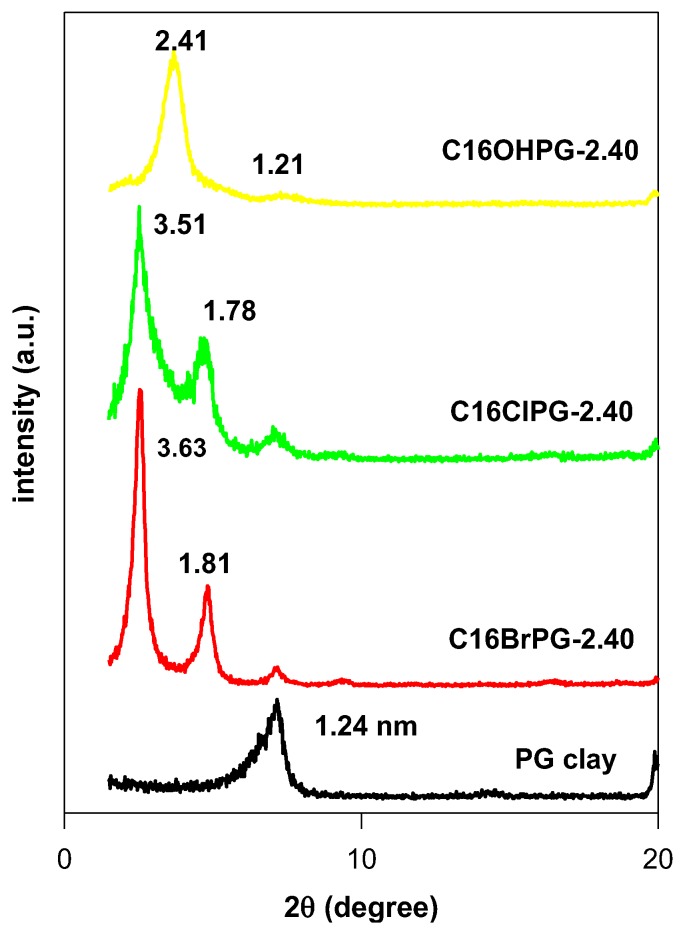
Powder XRD patterns of PG clay and its OCs prepared from different C16TMAX solutions.

The powder XRD patterns of the starting PG clay and its organo-derivatives prepared from different surfactant solutions at a fixed initial loading concentration of 2.4 mmol/g of clay (basis) are depicted in Figure 3.

The PG clay exhibited a 001 reflection at a basal spacing (d_001_) of 1.24 nm, which indicated that the raw sample is Na-montmorillonite with a monolayer of water molecules between the clay sheets [38,39]. After modification with surfactant solutions, a shift of the 001 reflection was observed to lower 2theta values and confirmed the intercalation of the surfactant cations in thePG interlayer spacing. The values of d_001_ increased from 1.24 nm to 3.63 nm and 3.51 nm when using C16TMABr and C16TMACl solutions. However, a lower value of 2.41 nm was obtained from a C16TMAOH solution. This observation indicated that the variation of d_001_ values was related to different contents of C16TMA cations, which is mentioned in Table 1 [31,35].

Many attempts were performed to increase the basal spacing (d_001_ value) of C16OHPG-2.40 by using different initial loading concentrations for lower concentrations than 2.40 mmol. No variation of the basal spacing was observed and even at higher concentrations close to 20 mmol (Figure S1), similar data were obtained for another clay mineral with lower CEC value (0.96 meq/g) [40]. However, using an acid activated clay mineral, a basal spacing of 3.83 nm was achieved at the same loading concentration of 2.40 mmol. This difference was related to different mechanisms of C16TMA intercalation [22].

**Figure 4:**
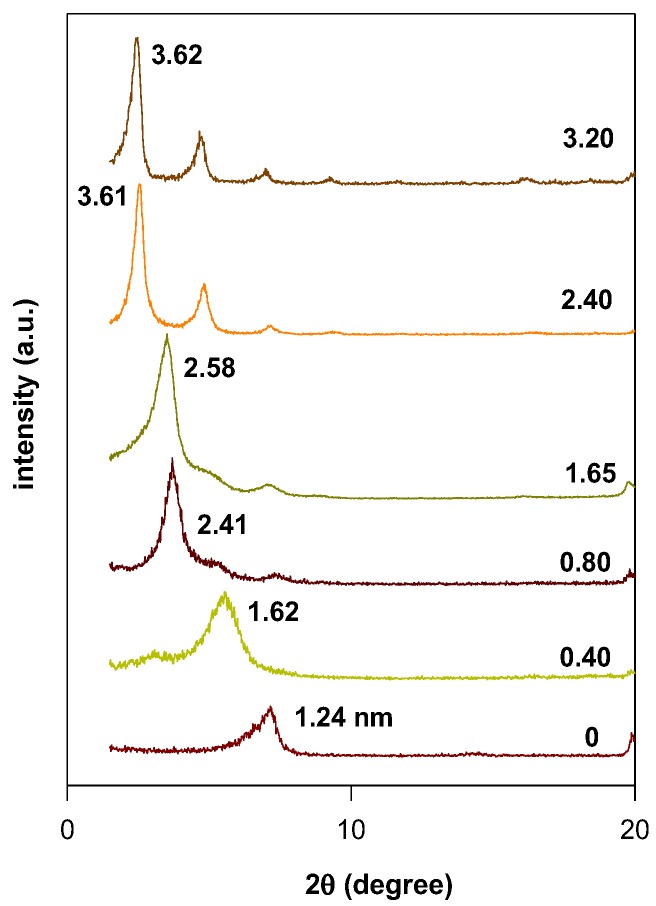
Powder XRD patterns of different OCs prepared from PG clay and C16TMABr solution at different initial loading concentrations (mmol/g).

The basal spacing of the OC prepared from C16TMABr solution was tuned by changing the initial loading concentrations. Figure 4 shows the PXRD patterns of the obtained materials.

The basal spacing of PG clay expanded gradually from 1.24 nm to 1.62 nm and 2.41 nm for initial concentrations lower than 2.40 mmol. Only one phase was obtained with d_001_ of 3.61 nm for C16TMA contents higher than 2.40 mmol. No further change of this value was noticed with regard to further increase of initial loading concentrations. Similar behavior was observed when the PG was modified with the C16TMACl solution, which is a significant increase of the basal spacing from 2.47 nm to 3.49 nm at initial concentrations greater than 2.40 mmol. This variation was due to different orientations of C16TMA cations between the clay sheets. The changes in orientation of the intercalated C16TMA cations was related to the initial loading concentrations and, thus, to the up-take (real) amount intercalated between the clay sheets, as presented in Table 2.

**Figure 5:**
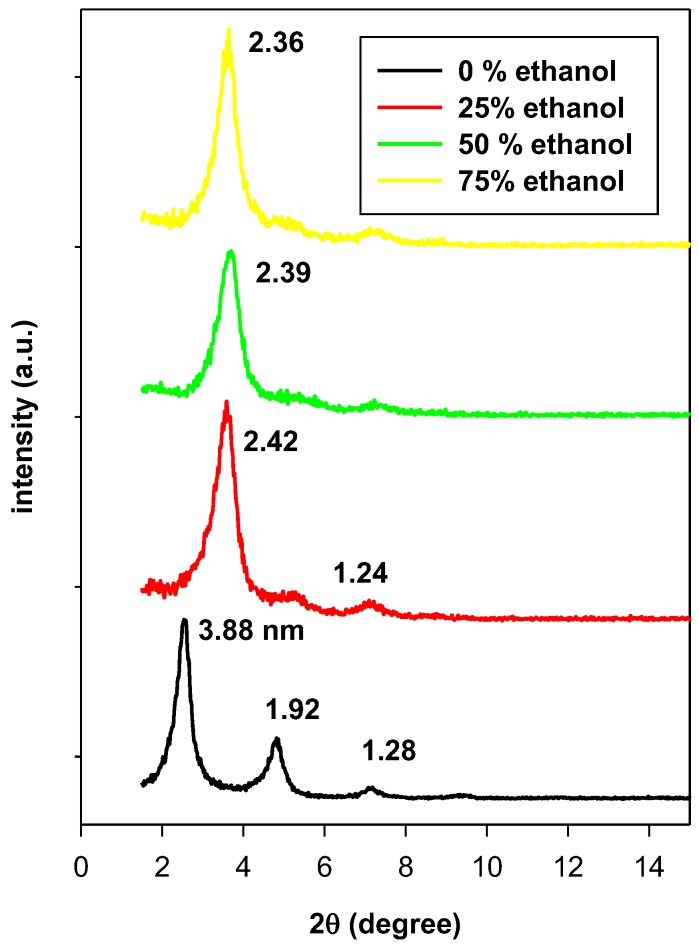
PXRD patterns of C16BrPG-2.40 washed with different aqueous ethanolic solutions (volume%).

However, this orientation was also related to the used exchange medium or to the washing solution [23]. Figure 5 presents the PXRD patterns of C16BrPG-2.40 washed with different ethanolic solutions (mixtures of ethanol/water in volume). The initial basal space of 3.88 nm was obtained during the washing process with pure deionized water. However, it shrank to 2.42 nm when it was washed with a mixture of 25% of ethanol and 75% of water. As the amount of ethanol increased in the washing solution, the position of the main peak practically does not change and the basal spacing was in the range of 2.39 nm to 2.36 nm.

These data were in good agreement with the C, H, N elemental analysis. The decrease in the basal spacing was related to a change in the surfactants configuration between the clay sheets. However, the effect of the washing solution was not noticed for the sample with low C16TMA content. A slight shrinkage was observed, which indicates the stability of the surfactant orientation between the clay sheets. Similar observations were noticed for an OC prepared from acid-activated clay made from C16TMAOH solution with a basal spacing of 3.83 nm [21]. The decrease of the basal spacing was proposed to be related to the dissolution of the physically adsorbed surfactants and to rearrangements of the intercalated surfactants [41] or it could be related to a partial dissolution of surfactants located between the clay sheets due to the easy accessibility of the ethanol molecules to these surfactants [21].

**Figure 6:**
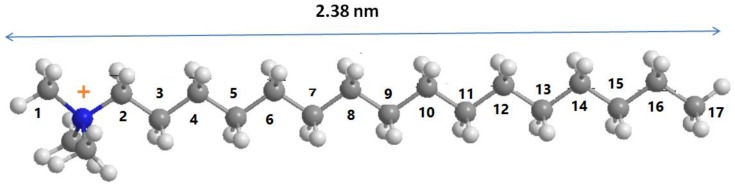
The optimized three-dimensional structural formula of the C16TMA cation.

From the basal spacing values (deduced from d_001_ value) and the thickness of the silicate layer of 0.96 nm [42], the corresponding interlayer spacing are summarized in Table 3. The structure of C16TMA cations could be ascribed as a nail with the trimethyl ammonium as the head and the C16 aliphatic chain as the tail with a length of 2.38 nm [43] (Figure 6).

**Figure 7:**
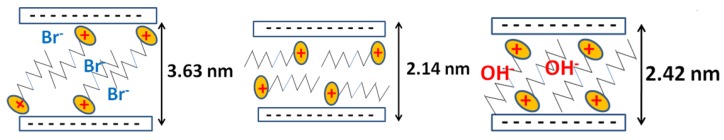
Illustrations of the structure of different organo-clays.

The interlayer spacing of 2.55 to 2.65 nm correspond to a bilayer arrangement of C16TMA cations tilted of an angle of 32° to 34°(degrees) relative to the surface of the clay sheets. The interlayer distance of 1.46 nm corresponds to a monolayer with a tilted angle of 35°. Lan et al. [44] proposed a relation between the chain length of the alkylammonium ion and the basal spacing as d_001_ = 1.27 × (n − 1) ‏+ d_A_ ‏+ r_M_. Where (n − 1) is the number of methylene groups in the onium ion chain, d_A_ is the basal spacing for NH_4_-montmorillonite (1.28 nm), r_M_ is the van der Waals radius of the methyl end group (0.3 nm), and 0.127 nm is the contribution due to the –CH_2_– chain segments when the chain adopts an *all-trans* configuration. In this case, the d_001_ would be 4.22 nm for the bilayer paraffin rearrangement. This value was higher than the observed one (3.61 nm), which indicates that the C16TMA cations did not adopt a vertical *all-trans* configuration. However, a lateral bilayer of C16TMA cations was obtained when a mixture of ethanol/water was used as the washing solution due to lower content of C16TMA cations in this sample. Conceptual illustrations for the structure of organs-clays are depicted in Figure 7.

### 3.3. Solid ^13^C CP NMR Studies

**Figure 8:**
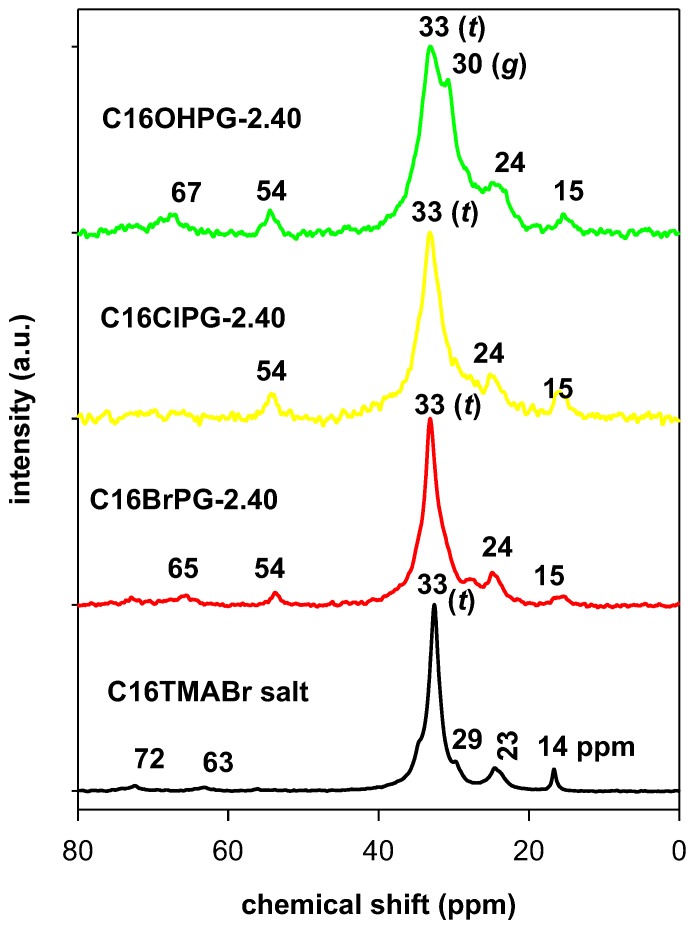
^13^C CP NMR spectrum of pure C16TMABr salt and the OCs prepared from different C16TMA solutions.

This technique is a powerful technique and it was used to probe the local environment and the conformation of the C16TMA cations between the interlayer spacing [45,46]. The ^13^C CP MAS resonance is sensitive to the difference in conformation and packing in addition to the chemical structure [47,48]. Figure 8 depicts the resonance spectra of the starting C16TMABr salt and OCs prepared from different solutions. The resonance spectrum of C16TMABr salt exhibits mainly one intense resonance peak at 33 ppm, which is assigned to the inner methylene (C_3_–C_16_) units of C16TMA (Figure 8) indicating that the methylene carbons exhibited *all-trans* (*t*) conformation [22] in addition to other resonances related to the C16TMABr salt [23] (see Table 4).

The ^13^C CP NMR spectrum of the OC prepared from C16TMA hydroxide solution (C16OHPG-2.40) exhibited a resonance at 30 ppm in addition to 33 ppm one, corresponding to the disordered and the ordered conformation, respectively [49] (Figure 8). Similar spectrum was reported for OC prepared from different clay minerals from the same C16TMAOH solution [21,22], the other resonance peaks and their assignments are reported in Table 4. The presence of the two resonance peaks indicated that the coexistence of *all-trans* (*t*) and *gauche* (*g*) conformations and confirmed the formation pseudo trilayer arrangement of the C16TMA cations or the paraffin monolayer arrangement between the clay sheets. The lateral bilayer arrangement gave rise to more *gauche* (*g*) conformation compared to *all trans* (*t*) one [47].

Qualitatively, the intensity of (*g*) conformation decreased dramatically when the C16TMACl and C16TMABr solutions were used and the ^13^C CP NMR exhibited mainly an intense resonance peak at 33 ppm similar to that of the pure C16TMABr salt, which is presented in Figure 8. The resonance peak at 30 ppm was embedded in the intense peak as a shoulder and indicated that the C16TMA cations exhibited mainly *all trans* (*t*) conformation due to the bilayer paraffin arrangement as indicated by the XRD technique [50]. In this arrangement, the trimethyl ammonuim heads were attached to the clay layers’ surfaces, which induces a shift of the resonance peak at 16 ppm, and the tails of the surfactants were pulled away. This gives rise to the *all trans* (*t*) conformation.

The estimated areas at the resonance peaks of 30 and 33 ppm after deconvolution operation in the range of 24 ppm to 50 ppm were used to probe the disordered degree of indicated C16TMA cations (Figure S2) [49,51]. The fraction of *gauche* conformation (*g*) was calculated as: *g*/(*g*+ *t*) = A*_g_*/(A*_g_*+ A*_t_*) where A*_g_* and A*_t_* are the integrated areas of the *trans* and *gauche* resonance peaks around 30 and 33 ppm. The fraction of *gauche* (0.15) was the lowest for the neat C16TMABr salt due to its crystalline order, which is supported by XRD data (see below). Then it increased for the intercalated surfactants with a maximum value of 0.48 for C16OHPG-2.40 sample and intermediate values of 0.38 and 0.24 for C16BrPG-2.40 and C16ClPG-2.40, respectively. The decrease of the *gauche* conformation was related to the transition of a liquidlike structure to semi-crystalline one with an increasing population of *trans* conformation [49].

### 3.4. Microtextural Properties

Table 3 summarizes the specific surface area (S_BET_), pore volume, and average pore diameter of the starting PG clay and selected derived OCs. The PG clay exhibited a S_BET_ value of 67 m^2^/g close to that reported for similar clay minerals [53]. The organoclays exhibited a lower S_BET_ in the range of 11 to 28 m^2^/g. These values were close to that reported for similar materials [25,53,54] (Table 5).

The average pore size in the OCs was higher when compared to the starting PG-clay. This enhancement was related to the repulsion between the organo-clay particles. Qualitatively, the increase of the A.P.D. value was related to the *gauche* fraction conformation. The total pore volume (P.V.) of PG clay decreased after modification [54]. Most commonly, the cationic surfactant head groups carry positive charge and are tightly bonded to the clay surfaces. Consequently, all cationic surfactants are expected to cover some/all of the mineral surfaces and decrease the apparent surface area of the surfactant/clay hybrid. Cationic surfactant head groups reduce the inter-particle repulsive forces, can cause particles to aggregate, and, therefore, can also reduce the surface area [51,54,55,56].

### 3.5. Thermogravimetric Analysis

**Figure 9:**
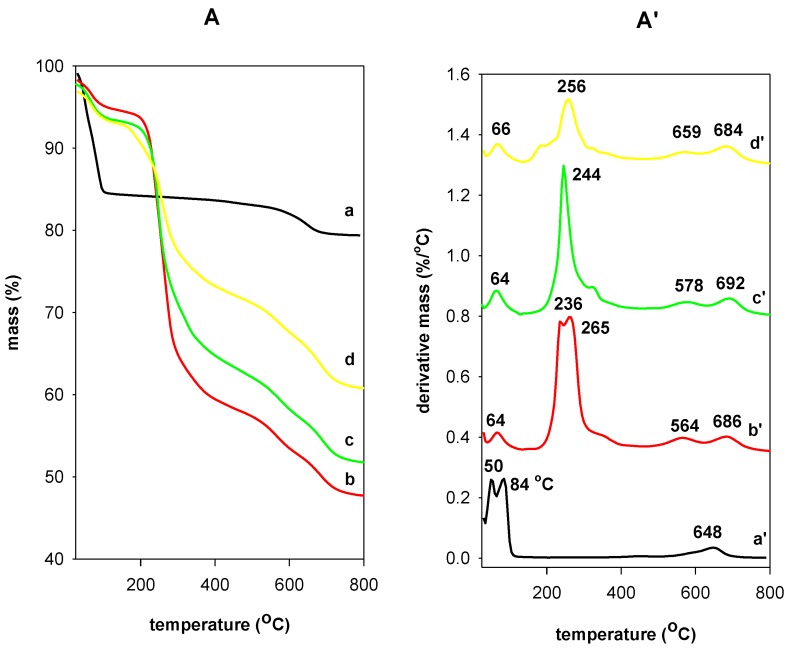
TGA (**A**) / DTG (**A’**) features of (a, a’) PG modified with different C16TMA solutions at initial loading of 2.40 mmol/g. (b, b’) C16TMABr, (c, c’) C16TMACl and (d, d’) C16TMAOH.

This technique was used to investigate the thermal stability of the OCs. The derivative thermogravimetric analysis (DTG) was performed to identify in more accuracy the temperature ranges on which the mass loss step occurs. The TGA/DTG features are presented in Figure 9. From the TG analysis, thermal decomposition of PG clay took place mainly in two steps while the thermal decomposition of the studied OCs occurred in three steps.

The PG clay exhibited a single mass of 15% from room temperature to 150 °C due to the removal of adsorbed water on the external surface and the dehydration of water molecules coordinated to Na^+^ cations, which are associated with two peaks at 50 °C and 84 °C, respectively [57]. A second mass of 3.7% at higher temperature ranges from 500 °C to 700 °C was attributed to the dehydroxylation of the clay sheets with a maximum temperature loss of 648 °C [57] (Figure 9). The OC (C16BrPG-2.40) exhibited additional mass loss that began at 170 °C (from DTG curve), which indicates the starting and the progressive elimination of the organic part in OC, and it ended at 400 °C with a maximum decomposition rate temperature of 256 °C [57]. From 428 °C to 800 °C, another mass of 10.4% was observed and associated to the dehydroxylation of the clay sheets and the progressive burnout of the residual carbonaceous materials [58] (Figure 9). The mass related to water molecules was reduced to 4.6% and was related mainly to physisorbed water molecules on the surface with a peak on the DTG curve at 64 °C [57,58]. The mass related to coordinated water molecules to Na^+^ cations vanished due to their exchange with C16TMA cations [59]. A similar feature was obtained for the OCs prepared from C16TMACl and C16TMAOH solutions (Figure 9). However, the area of the DTG peak related to the organic mass was lower, which indicated a lower organic content in the C16OHPG-2.40 clay. This fact was in good agreement with C.H, N analysis. The maximum temperature mass related to the loss of organic matter (256 °C) was higher for C16OHPG-2.40 compared to the other OCs at 244 °C and 236 °C, which is presented in Figure 9. The variation in the temperature reflected the easiness to loss organic matter with higher contents due to the high expansion of the interlayer spacing and it was easy to remove them. However, with low content of organic matter, the basal spacing was not expanded sufficiently and the cations adopted a lateral monolayer arrangement, which made their removal between the clay sheets difficult. The decrease in the dehydration peak of DTG curves was related to the conversion of PG clay to the organophilic character.

The different mass steps were accompanied with different events in the DTA curves. Figure S3 depicts the DTA features of PG clay and the OCs prepared from different C16TMA solutions. The loss of different types of water molecules from PG clay was associated to two endothermic peaks at 57 °C and 90 °C, respectively. However, in case of OCs, only one type of water molecule was lost at 65 °C. Additional exothermic peaks were observed at mainly 235 °C and 373 °C with shoulders at 580 °C and 690 °C. These peaks were associated with the combustion of the organic surfactants and their residue carbon materials. The dehydroxylation of the OCs was difficult to detect when compared to the starting PG clay, which is associated with a broad endothermic peak at 650 °C [60].

From Table 6, the general trend related to water content could be noticed. The water content decreased in the OCs, which confirms that the Na^+^ cations were fully exchanged and the OCs exhibited an organophilic character. The organic content depended on the used C16TMA solution. The experimental content was close to the theoretical values deduced from the C, H, N analysis.

The C16TMABr salt exhibited one peak in the DTG feature at 240 °C, which indicates that decomposition occurred cleanly in one single step with almost negligible residual mass (not shown). This value was lower to that reported for pure and recrystallized C16TMABr salt (268 °C) [61]. The maximum decomposition temperature rate of the intercalated C16TMA cations occurred at higher temperatures when compared to the pure C16TMABr salt. This difference was related to the effect of the clay layers on the decomposition of the surfactants. However, in case of pure layered silicate materials that do not contain aluminum in their structures, the decomposition occurred at lower temperatures. The residues at 900 °C are 47.52% for C16BrPG-2.40, 53.56% for C16ClPG-2.4, and 63.56% for C16OHPG-2.40. These were also distinctive, which confirms the structural modifications of the clays even though they retained their inorganic character.

**Figure 10:**
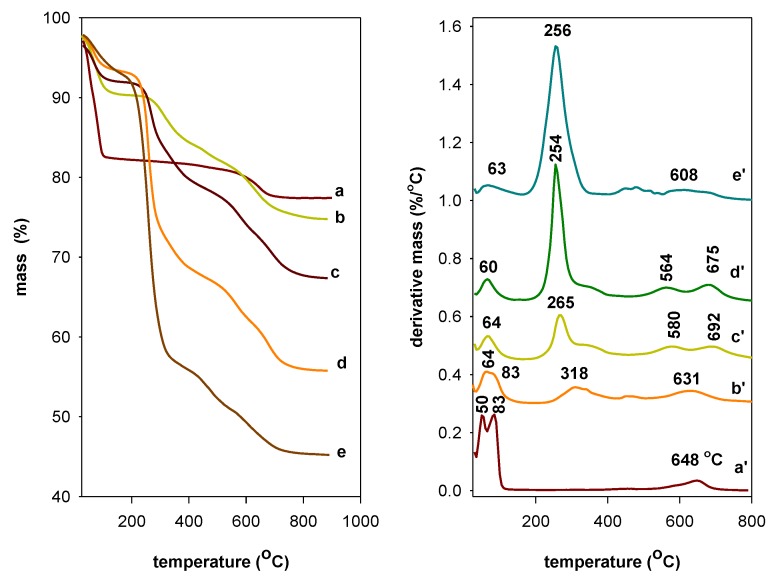
TGA (**left**)/DTG (**right**) curves of (a, a’) PG clay modified with C16TMAbr solution at different concentrations (b, b’) 0.40 mmol, (c, c’) 0.80 mmol. (d, d’) 1.65 mmol and (e, e’) 2.40 mmol.

For OCs prepared at different initial concentrations from C16TMABr solution, the TGA/DTG features are depicted in Figure 10, and the different mass percentages are summarized in Table 7. As the content of C16TMA cations increased in the synthesized materials, the mass related to water molecules decreased from 15.54% to 3.72% and only one type of water molecules was removed at a temperature of 60 °C to 64 °C (as indicated by the DTG peak) in addition to the area of this peak, which was also decreasing. At the same time, the area of the peak related to C16TMA cations was improved when the initial loading concentration rose. We noticed that the related maximum temperature peak was shifted to lower values. This shift indicated that, at higher organic content in the OCs, the C16TMA cations were easy to remove because of their particular arrangement in the interlayer space, which was not the case of lower C16TMA content. They exhibited different arrangements (with basal spacings of 2.14 and 2.51 nm) and, per consequent, difficult to remove in good agreement with the reported data above.

### 3.6. Thermal Stability Study

As mentioned in the experimental part, the in-situ XRD technique was performed to identify the appropriate temperature at which the OCs could be treated without losing their performance in the eosin removal process [25].

**Figure 11:**
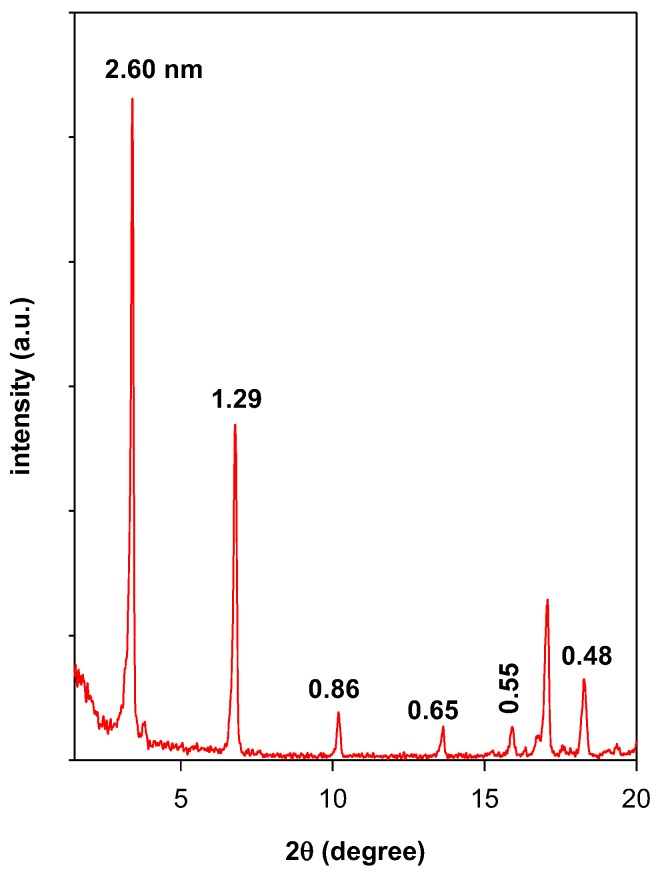
Powder XRD pattern of the starting C16TMABr salt.

The Powder XRD pattern of C16TMABr salt consists of a multiple discrete peaks characteristic of a crystalline state and lamellar structure with a very sharp strong peak corresponding to a repeat ranging of 2.60 nm (Figure 11) [62]. The value of 2.6 nm was estimated from the position of the first five reflections.

**Figure 12:**
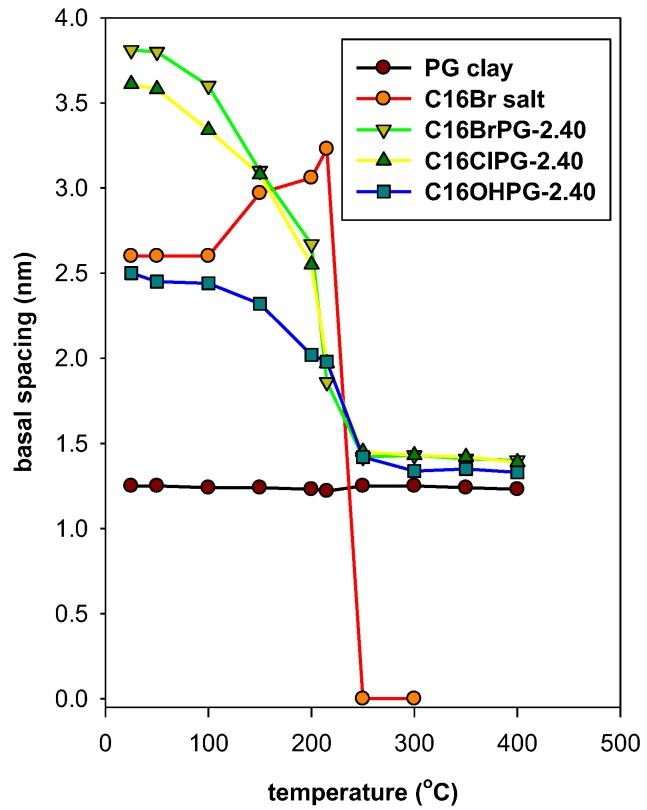
The variation of basal spacing (d_001_) of different materials with preheated treatment temperatures.

After preheating at different temperatures, the powder XRD patterns are presented in Figure S5. The variation of the distance related to 001 reflection (d_001_) is summarized in Figure 12. The layered structure of C16TMABr was maintained at temperatures of 215 °C with a gradual increase of the d_001_ from 2.61 nm to 3.23 nm. Then no reflection was detected due to the melting of the C16TMABr salt [25].

The in-situ study of organo-clay (C16BrPG-2.40) indicated that the expanded form of the organo-clay was maintained at temperatures lower than 150 °C.Then it collapsed to a value of 1.86 nm when treated at 215 °C. The basal spacing continued to shrink as the temperature increased due to the progress of the remaining carbon materials (Figure 12). Similar in situ data were obtained for the C16ClPG-2.40 and indicated that the expanded structure was maintained at temperatures lower than 200 °C. The basal spacing values were in the range of 3.61 to 3.0 nm. The chain of the carbon moiety was decomposed at 215 °C, which results in a decrease in the basal spacing of the organo-clay to 2.10 nm. The intercalated C16TMA cations behave differently than the neat salt in the way that there was no increase of the basal spacing of the OCs in the range of 100 to 150 °C, which was reported for the C16TMABr salt. However, when other types of silicate were used as a host matrix, the intercalated C16TMA cations behave in the same way than the C16TMABr salt [25,63]. This fact could be related to an intrinsic effect of the type of silicate layers and its properties.

In case of OC prepared from C16OH solution (C16OHPG-2.40), the arrangement of the C16TMA cations was different than that of C16TMABr and C16TMACl solutions. The decrease of the basal spacing will occur at relatively higher temperatures, which is indicated by the TGA technique. The expanded organo-clay was maintained to temperatures up to 150 °C with a slight variation of the basal spacing. Then, it collapsed to 1.43 nm due to the combustion of the organic C16TMA cations, which was presented in Figure 12.

For all the materials, the basal spacing of treated materials (1.43 nm) at temperatures higher than 250 °C was higher than the basal spacing of the pristine PG treated at the same temperature (1.24 nm). This variation was related to the presence of the residual carbon materials between the clay sheets [25,34]. These data are in good accordance with the TGA data, which was presented in Table 4.

### 3.7. Removal Properties of Eosin Dye

#### 3.7.1. Effect of the Initial Concentration

The removal of eosin was investigated by varying the initial concentrations (C_i_) of the dye solution in the range of 25 mg/L to 1000 mg/L for the C16BrPG-2.40. The removal efficiency decreased from 100% to 85% since C_i_ of eosin increased from 25 mg/L to 1000 mg/L (Figure S5). The reduction in eosin indicated that the available removal sites become lower at higher C_i_ values since the mass of the OC was kept constant. However, the removal amount of the eosin increased from 2.5 mg/g to 76.5 mg/g since the initial eosin concentration increased from 25 mg/L to 1000 mg/L (as shown in Figure S5). At lower eosin initial concentration, the ratio between the numbers of eosin molecules to the number of available removal sites is low and the rate of diffusion of eosin molecules was slow. Hence, few dye molecules reach adsorption sites. However, a higher initial concentration provided a higher driving force for eosin molecules to approach removal sites more rapidly. Moreover, the number of collisions between dye molecules and adsorbent increases and improves the removal process [64,65].

#### 3.7.2. Effect of C16TMA Contents

**Figure 13:**
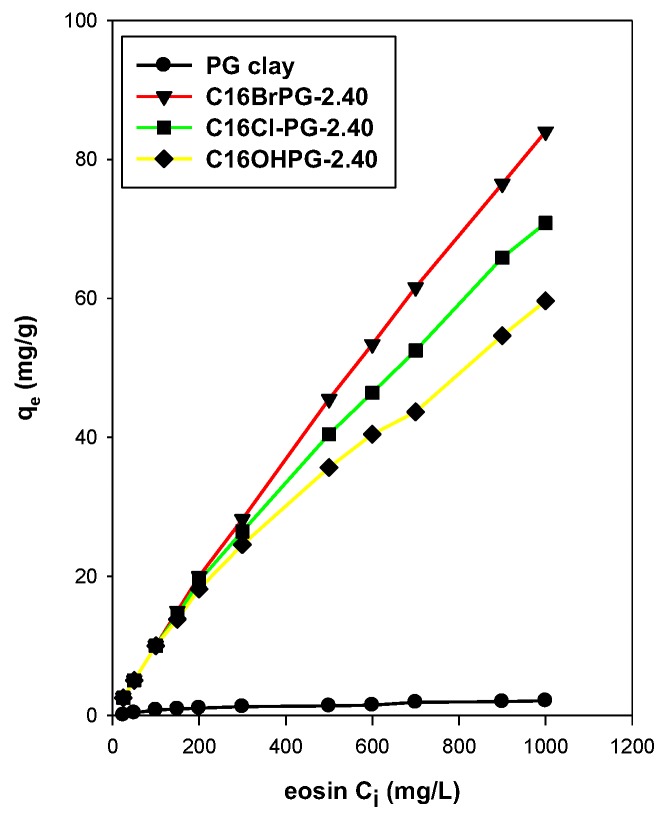
Removal of eosin at different initial concentrations by different OCs.

Figure 13 shows the effect of the amounts of C16TMA contents on the removal capacities of eosin onto different OCs. As expected, the PG clay exhibited very low removal capacity due to the repulsion between the negative electrical charge of PG clay and eosin dye molecules [16]. Similar observations were noted for other silicate layered materials such as kenyaite and magadiite [25,65].

Figure 13 indicated that low removal capacities were obtained for low loading of the C16TMABr amount because of the remaining negative electrical charge on the PG clay surface [32]. As the C16TMA content increased, the removal amount was also improved due to the complete covering of the negative charge on PG clay by the surfactant, which means the electrical repulsion overcame [66,67,68]. Similar data were achieved using local clay mineral [32] and other silicate layered materials [25,63].

**Figure 14:**
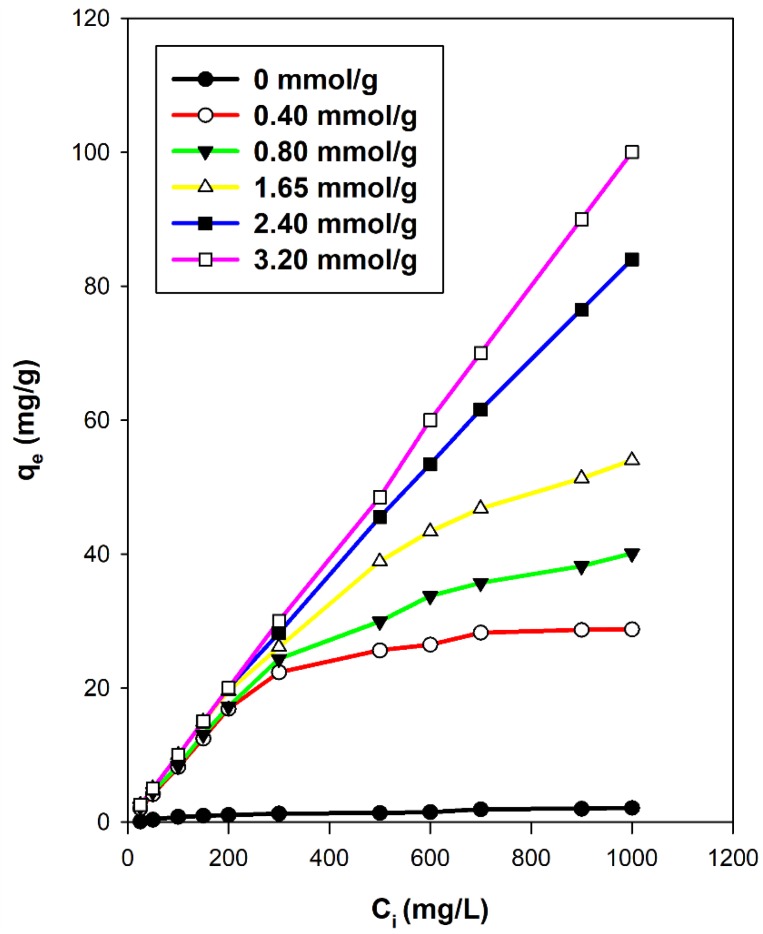
Removal of eosin using organo-clay prepared from C16TMABr solution using different initial loading concentrations.

Figure 14 presents the removal efficacy of OCs prepared from the C16TMABr solution at different initial loading concentrations. At C_i_ values lower than 200 mg/L, the removed amount was independent of the amount of C16TMA cations in the OCs. At higher C_i_ values greater than 200 mg/L, the removed eosin was improved as the C16TMA contents increased in the OC materials. This part indicated that the modification of the PG clay with surfactants has enhanced the removal of an acidic dye. Similar data were obtained using different organo-silicates [25,63].

The modification of the silicate surface by organic cations and mainly long ones such as C16TMAs rendered the PG clay an organophilic material [57]. The negatively charged surface of the silicate may adsorb the C16TMA^+^ cations through an ion exchange mechanism where a monolayer of cationic surfactants on the surface of the clay formed. The positively charged ends of the cationic surfactants were exchanged with the exchangeable interlayer cations of the PG clay (Na^+^) and the hydrophobic head of the cationic surfactants was arranged outward [57]. The C16TMA^+^ cations generated an organophilic phase partition in the interlayer spacing and the partition occurred through the interaction of the dye with the cationic C16TMA^+^ cations [65,66,67,68]. The removal amount was improved with the increase of eosin C_i_ values independently of the used ogano-clay and the maximum value (84 mg/g) was obtained at C_i_ of 1000 mg/L using C16BrPG-2.40.

It is observed that the organo-clays exhibited lower specific surface areas. However, the amount of removed eosin remained high, which suggested that the eosin molecules were removed into the interlamellar space. The PXRD patterns after the removal of dyes indicated a slight variation of the basal spacing and could indicate an anion exchange of Br anions with the removed eosin dyes [69]. 

#### 3.7.3. Effect of Temperature

The temperature has important effects on the removal process [66]. Three different temperatures (25 °C, 35 °C, and 50 °C) were studied. The temperature of 50 °C was selected because no change in the OC structure occurred. It was observed that, as the experimental temperature increased, the removed amounts of eosin by the used OCs was improved and it varied from 84 mg/g to 110 mg/g (Figure S6). This suggests that the removal process is favored at higher temperatures. It is common that increasing the temperature may create a swelling influence inside the OCs in the eosin solution structure and additional eosin dye molecule penetrate the removal sites [70].

The distribution coefficient is an important parameter for estimating the affinity of OC towards eosin in aqueous solution. The quantity *K_d_* may be defined by Equation (1).

(1) $$K_{d}=\frac{C_{e}}{C_{a}}$$

where *C_e_* is the equilibrium concentration (mol/L) and *C_a_* is the amount of dye adsorbed on the adsorbent at equilibrium (mol/L) [71]. The increase in temperatures was associated with an enhancement of the affinity of OC towards eosin, which is indicated by the increase of *K_d_* values (Table 8).

The thermodynamics parameters were estimated using Equations (1)–(3) [71] and are presented in Table 8.

(2) $$\Delta G{}^{\circ}=-RT\ln K_{d}$$

The thermodynamics parameters were also estimated by using the Van’t Hoff equation.

(3) $$LnK_{d}=\frac{\Delta S{}^{\circ}}{R}-\frac{\Delta H{}^{\circ}}{RT}$$

where *R* is the gas constant (J mol^−1^ K^−1^), Δ*G*° is the free energy (kJ mol^−1^), *K_d_* is the distribution constant, T is the absolute temperature (K), Δ*H*° is the standard enthalpy (kJ mol^−1^), and Δ*S*° is the standard entropy (kJ mol^−1^ K). Δ*S*° and Δ*H*° values were achieved from the intercept and slope of plot ln *K_d_* versus 1/T.

These parameters provide details on the spontaneity, feasibility, and eosin-organo-clay interactions during the removal process. Figure S7 presents the Van’t Hoff plot and exhibited alinearity. From Table 8, the removal of eosin was an endothermic process with enthalpy (Δ*H*°) being 41.22 kJ/mol. An increase in negativity of the Gibbs free energy (Δ*G*°) with the temperature suggested improved spontaneity of the eosin-OC system at an elevated temperature. The entropy value of Δ*S*° (0.153 KJ/mol) indicated that the eosin removal by OC leads to the randomness in the solid/liquid interface and suggested an increase of the disorder at the eosin-OC interface [72].

#### 3.7.4. Effect of Preheated Treatment of OCs

For the convenience purposes, only two samples of OCs were selected in this part: C16BrPG-2.40 and C16OHPG-2.40. As mentioned above, these samples had different organic contents.

**Figure 15:**
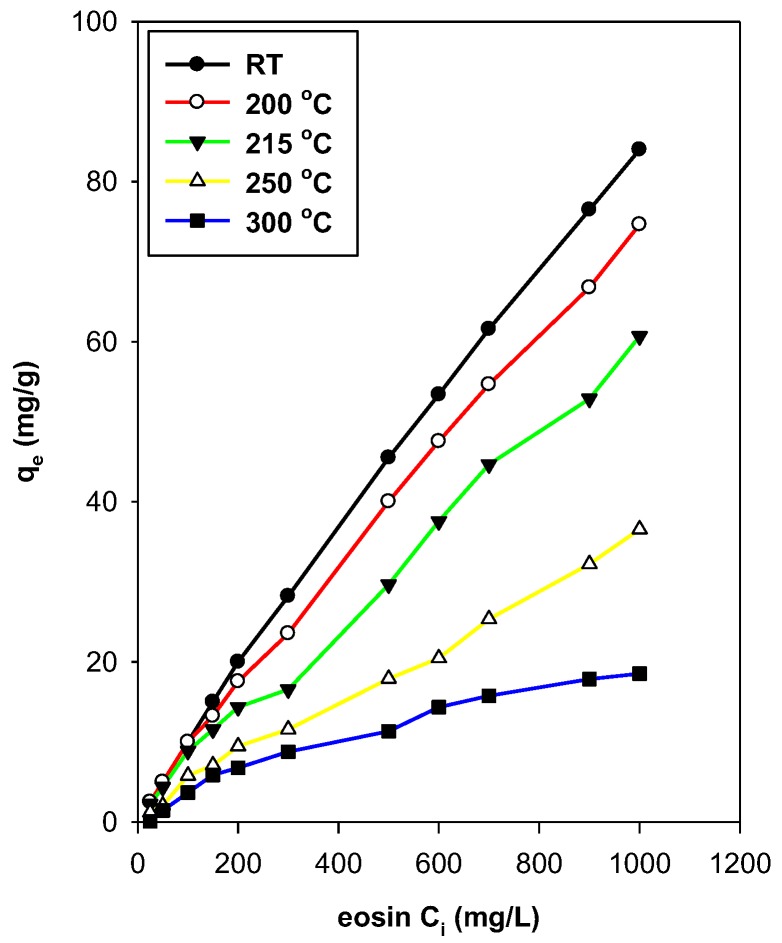
Removal capacity of C16BrPG-2.40 preheated at different temperatures.

Figure 15 presents the evolution of the removed amount of eosin as it functions in the preheated temperatures of the C16BrPG-2.40 in the C_i_ range of 25 to 1000 mg/L. At C_i_ values less than 200 mg/L, the removed amount was independent of the preheated temperatures less than 150 °C. However, it was reduced when C16BrPG-2.40 preheated at temperatures higher than 200 °C and C_i_ values higher than 200 ppm due to the beginning of the decomposition of C16TMA cations, which was deduced from TGA and the in situ studies. Preheat treatment at temperatures higher than 230 °C resulted in a reduction of removed eosin amounts due to the complete breakdown of the intercalated C16TMA cations (as indicated by the in situ XRD studies). Per consequent, a destruction of the active sites responsible for the removal of eosin occurred. The clear effect of preheat treatment was observed at higher C_i_ value of 300 to 1000 mg/L.

The C16OHPG-2.40 exhibited similar behavior, which indicated that the removal properties were maintained at heating temperatures higher than 150 °C for lower C_i_ values than 200 mg/L (Figure S8). However, less extension is compared to C16BrPG-2.40 due to the lower contents of C16TMA in the C16OHPG-2.40. The removed amount of eosin was reduced at preheated temperatures higher than 200 °C due to the decomposition of C16TMA cations and due to the loss of the removal sites. The decrease was also observed for higher concentrations of 1000 mg/L.

Similar data were reported for similar silicate layers such as kenyaite and magadiite [25,63]. Nevertheless, the preheated treatment may help find the optimal temperature at which the modified OCs could be used. The preheated temperatures were reported to affect the adsorption properties of nitrobenzene too [73]. Compared to PG clay, the preheated OCs at temperatures higher than 250 °C still exhibited minor removal properties due to the remaining sites.

### 3.8. Maximum Removal Amount

The Langmuir equation was used to estimate the maximum removed amount (*q*_m_). This equation was based on the assumption that a monolayer is adsorbed and covers all the available surface of the adsorbent [74]. The linearized Langmuir isotherm allows for the calculation of the adsorption capacity (*q*_max_) and the Langmuir constant (*K_L_*) that are equated by Equation (4).

(4) $$\frac{C_{e}}{q_{e}}=\frac{1}{q_{\max}\cdot K_{L}}+\frac{C_{e}}{q_{\max}}$$

where *C_e_* and *q_e_* are the concentration at equilibrium (mg/g) and the amount adsorbed at equilibrium (mg/g), respectively. These constants can be estimated from the intercept and slope of the linear plot of the experimental data of *C_e_*/*q_e_* versus *C_e_*.

As mentioned above, the modification of PG clay with C16TMA cations has improved its removal capacities (Table 9). The maximum value of 90.90 mg of eosin/g of OC was achieved for the C16BrPG-2.40 as starting material. However, the lowest value of 60.60 mg/g was attained using C16OHPG-2.40. The removal efficiency was dependent on the preheated treatment. It was maintained at temperatures below 210 °C. Then it was decreased significantly. This fact was related to the destruction of the intercalated C16TMA cations and, thus, there was no availability of removal sites in this process (Table 9). The *K_L_* (parameter of the Langmuir model) is a measure of the affinity between the esoin dye and the organo-clays. In other words, it expresses the binding constant of eosin and the organ-clay. Since the binding is based on the electrostatic interaction, the *K_L_* value increased when the removed amount was improved (Table 9). Similar trends were obtained for a different organo-silicates [25,63].

Compared to local clay mineral and other silicate materials, the OC prepared from PG clay exhibited higher removal efficiency due to the difference in organic contents (Table 10). These data indicated that the OCs derived from PG clay mineral could be considered as a potential candidate for dyes removal.

### 3.9. Regeneration Cycles

The removal of eosin by OCs concentrates the dye molecules by transferring them into other phases and these concentrated dye molecules cause another environmental disposal problem. A good removal agent will depend not only on its removal capacity but how well it can be regenerated and repeatedly used.

Different methods of regeneration were proposed in the literature including thermal treatments, Fenton oxidation, wet air oxidation, biological, ultrasound, electrochemical methods, and microwaves, etc. [77]. In this study, we used an environmentally-friendly method in the sense that it does not use a lot of chemicals and the solution could be used repeatedly many times without losing its efficiency [25,63].

**Figure 16:**
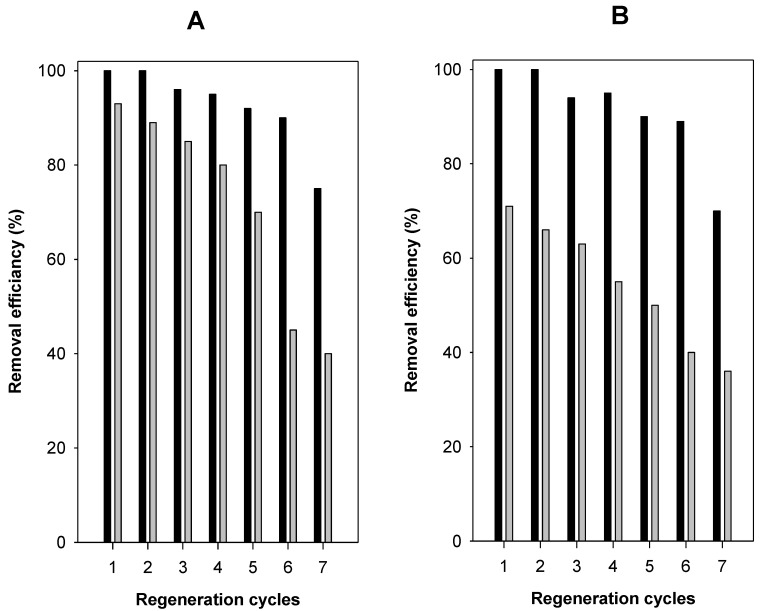
Regeneration efficiency of (**A**) C16BrPG-2.40 (B) C16OHPG-2.40 using C_i_ of 50 mg/L (dark bar) and 200 mg/L (grey bar).

The regeneration of C16BrPG-2.40 was investigated using two eosin C_i_ values of 50 mg/L and 500 mg/L. At lower C_i_ value of 50 mg/L, the removal efficiency was maintained till six cycles of re-use with slight variation of the removal percentage from 95% to 90%. Then it dropped to 7%. However, using high C_i_ value of 500 mg/L, the removal percentage of C16BrPG-2.40 was maintained after 4 removal/regeneration cycles and it varied from 93% to 80%. Then, it dropped to 45% after 6 cycles (Figure 16A). In case of C16OHPG-2.40 and for C_i_ of 500 mg/L, its efficiency was maintained for 3 cycles of regeneration and it varied from 71% to 63%. A significant drop was observed after the fourth cycle to 55% and it continuously decreased to 40% after the sixth cycle. For low C_i_ of 50 mg/L, the efficiency was maintained to the sixth cycle and varied from 100% to 90%. Then it dropped to 70% (Figure 16B). The decrease in the removal efficiency could indicate that some eosin anions were strongly attached to the removal sites, which makes it difficult to oxidize them by the used aqueous solution of oxone and Co^2+^ cations for the regeneration process [25,33].

## 4. Conclusions

The removal of anionic dye molecules "eosin" is weak at the PG clay mineral surface due to the negative charge of its surface. However, the modification with C16TMA cations has improved its removal efficiency and capacity. A maximum of 90.90 mg/g was achieved. The removed amount was controlled by the initial concentration, the operating temperature, and the preheat treatment of the OCs prior to their use.

The intercalated amount of C16TMA cations played an important role in the eosin removal and it could be tuned by varying the initial loading concentrations or choosing the right counter-ion in the starting solution. The C16TMABr solution has led to higher uptake of C16TMA cations (1.60 mmol/g), which is superior to the CEC value (1.45 meq/g). Apart from the C16TMAOH solution, the up take amount of organic surfactants was lower than the CEC value. The arrangement of C16TMA intercalated cations depended on the up take amount. Higher basal spacing of 3.82 nm was achieved for an uptake amount of 1.60 mmol/g, which is associated with a bilayer arrangement with a tilt angle of 32° on the clay sheets. ^13^C CP NMR indicated that the intercalated C16TMA cations exhibited mainly *all trans* conformation in addition to *gauche* conformation. The modified OCs exhibited an organophilic character deduced from the TGA data. The preheat treatment of two selected OCs affected their eosin removal properties at temperatures higher than 215 °C where the C16TMA cations were decomposed and accompanied by the loss in eosin removal efficiency. The regeneration process indicated that the removal property was maintained up to 6 cycles depending on the eosin C_i_ values. These data could indicate that the prepared OCs will be considered as a potential candidate to act as a removal agent for eosin dye molecules with a maximum of 90 mg/g.

## Figures and Tables

**Table 1:** C, H, N elemental analysis of different OCs prepared at different conditions.

Samples	C%	H%	N%	Up Take Amount (mmol/g) *
C16BrPG-2.40	39.23	5.88	2.34	1.60
C16ClPG-2.40	30.77	4.88	1.96	1.26
C16OHPG-2.40	18.91	3.60	1.69	0.93

* Up take amount = C (%)/[(12 × (number of carbon atom in CTMA)] × 1000.

**Table 2:** C, H, N analysis of C16BrPG-2.40 after washing with different mixtures of ethanol and water.

Samples	C%	H%	N%	Up Take Amount (mmol/g)
C16BrPG-2.40	39.23	5.88	2.34	1.60
25% ethanol *	20.52	4.40	1.22	0.90
50% ethanol *	19.55	4.87	1.06	0.85
75% ethanol *	18.76	4.54	1.01	0.82

* Washed with a mixture of ethanol and water (% in volume).

**Table 3:** The basal spacing values of OCs and possible arrangement of the intercalated C16TMA cations.

Uploaded Amount	d_001_ (nm)	Interlayer Spacing	Arrangement
0.40 (Br)	1.62	0.66	Lateral monolayer
0.80 (Br)	2.14	1.18	Lateral bilayer
1.65 (Br)	2.51	1.55	Pseudo-trilayer
2.40 (Br)	3.61	2.65	Paraffin bilayer, θ = 34°
3.20 (Br)	3.61	2.65	Paraffin bilayer, θ = 34°
C16BrPG-2.40r	3.61	2.65	Paraffin bilayer, θ = 34°
C16ClPG-2.40	3.51	2.55	Paraffin bilayer, θ = 32°
C16OHPG-2.40	2.42	1.46	paraffin monolayer, θ = 38° or pseudo trilayer
C16BrPG-2.40 *	2.42	1.46	Lateral bilayer

* Washed with Ethanol/water mixture (50% in volume).

**Table 4:** ^13^C chemical shift (ppm) of C16TMA in bulk and in OCs.

Carbon Atom	C16BrPG-2.40	C16OHPG-2.40	C16TMABr Solid
C_2_	65	67	63 (67.05, 64.55)
C_1_	54	54	53 (54.61)
C_15_	35	35	35 (36.40)
C_3_–C_17_ (*trans*, *t*)	33	33	33 (34.70)
C_3_–C_17_ (*gauche*, *g*)	30	30	29 (30.77–29.19)
C_2_, C_16_	24	24	23 (23.12)
C_17_	15	15	14 (16.14, 14.12)

Values between brackets are reported from Reference [52].

**Table 5:** Textural properties of PG clay and its organo-derivatives prepared from surfactant solution with different counter anions. The *g* conformation ratio was also presented.

Samples	S_BET_ (m^2^/g)	P.V. (cc/g)	A.P.D (nm)	(*g*) Ratio Conformation
PG-clay	67.0	0.147	8.79	-
C16BrPG-2.40	23.4	0.081	13.6	0.36
C16ClPG-2.40	28.4	0.078	10.9	0.24
C16OHPG-2.40	11.5	0.086	19.8	0.47

**Table 6:** Presents the water and the organic content in some selected OCs prepared from different surfactant solutions.

Samples	Water Content (Mass%)	Organic Content * (Mass%)	Residue at 900 °C
PG-clay	15.54	0	79.49
C16BrPG-2.40	3.51	36.13	47.52
C16ClPG-2.40	4.26	35.43	53.56
C16OHPG-2.40	3.85	18.79	63.56

* Mass in the range of 180 °C to 420 °C.

**Table 7:** Summarizes the mass related to water, surfactants molecules, and the residue from RT to 900 °C for samples prepared from C16TMABr solution.

Samples	Water Content (wt%)	Organic Content (wt%)	Residue at 900 °C
PG-clay	15.54	0	79.49
C16BrPG-0.40	7.49	6.36	77.14
C16BrPG-0.80	4.71	12.97	67.26
C16BrPG-1.65	4.22	26.17	57.94
C16BrPG-2.40	3.72	34.98	49.08

**Table 8:** Thermodynamic parameters of eosin removal using C16BrPG-2.40 organo-clay.

Parameter	Temperature		
	298 K	308 K	323 K
Δ*G*° (kJ mol^−1^)	−4.11	−6.02	−7.91
*K_d_*	5.25	10.49	19.00
Δ*S*° (kJ mol^−1^·K)	0.153		
Δ*H*° (kJ mol^−1^)	41.22		

**Table 9:** Langmuir parameters of the eosin removal by different OCs.

Samples	*q*_max_ (mg g^−1^)	*K_L_* (L g^−1^)	R^2^
PG clay	3.45	0.00054	0.9675
C16BrPG-2.40	90.90	0.0263	0.9934
C16ClPG-2.40	74.07	0.0154	0.9921
C16OHPG-2.40	60.60	0.0117	0.9886
C16BrPG-0.40	30.76	0.0223	0.9974
C16BrPG-0.80	46.20	0.0242	0.9873
C16BrPG-1.65	54.64	0.0653	0.9885
C16BrPG-3.20	94.20	0.1234	0.9921
C16BrPG-2.40 (100) *	75.11	0.0096	0.9914
C16BrPG-2.40 (150) *	75.11	0.0096	0.9934
C16BrPG-2.40 (200) *	64.47	0.0063	0.9827
C16BrPG-2.40 (215) *	53.76	0.0039	0.9854
C16BrPG-2.40 (250) *	38.09	0.0014	0.9875

* Preheated treatment temperature value in °C.

**Table 10:** Removal capacities of various adsorbents for eosin dye.

Samples	*q*_max_ (mg/g)	References
Organo-PG clays	75.11 to 90.90	[This work]
Organo-magadiites	69.54	[63]
Organo-local clays	48.66	[32]
Organo-kenyaites	48.01	[25]
Raw fly ash	43.48	[75]
Alumina nanoparticles	47.78	[76]

## Supplementary Materials

The following are available online. Figure S1: Variation of basal distance (d_001_) with the initial loading concentrations of C16TMAOH solution, Figure S2: Deconvolution of 13C CP MAS peaks in the range of 25 ppm to 45 ppm for C16TMABr salt and the OC materials, Figure S3: DTA curves of (a) PG clay and organo-clays prepared from different solutions (b) C16TMABr, (c) C16TMACl, and (d) C16TMAOH solutions, Figure S4: PXRD patterns of C16TMABr salt preheated at different temperatures (°C), Figure S5: Evolution of removed amount (mg/g) and removal percentage (%) using C16BrPG-2.40 organo-clay, Figure S6: Eosin removal properties of C16BrPG-2.40 organo-clay performed at different temperatures, Figure S7: The Van’t Hoff plot of eosin removal by C16BrPG-2.40 organo-clay at different temperatures, Figure S8: Removal capacity of C16OHPG-2.40 preheated at different temperatures and using two C_i_ values 200 mg/L (black) and 1000 mg/L (red).
