# Supplementary material for: Removal Properties of Anionic Dye Eosin by Cetyltrimethylammonium Organo-Clays: The Effect of Counter-Ions and Regeneration Studies
Source: Molecules. 2018 Sep 15;23(9):2364. doi: 10.3390/molecules23092364 (PMC6225147; doi:10.3390/molecules23092364)
Supplement: Supplementary file 1 [file molecules-23-02364-s001.pdf]

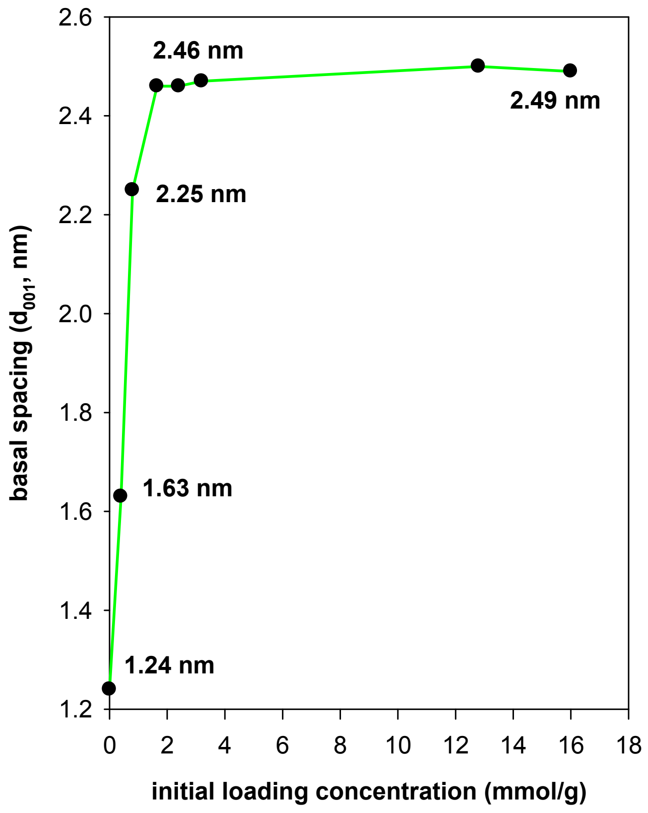

Figure S1. Variation of basal distance (d_{001}) with the initial loading concentrations of C16TMAOH solution.

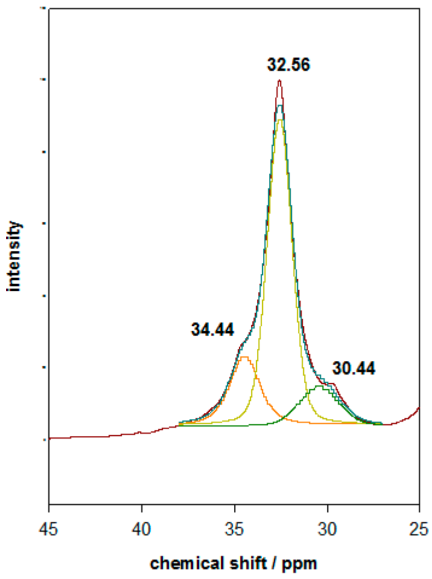

C16TMABr salt

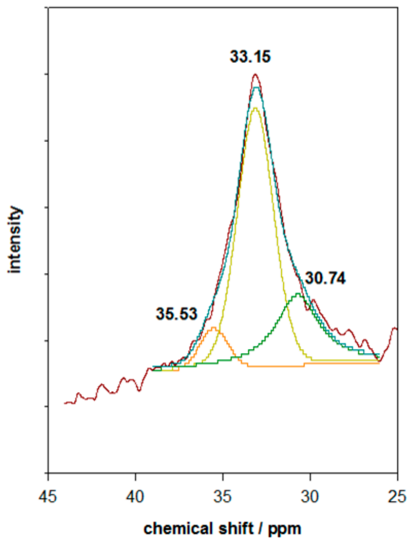

C16BrPG-2.40

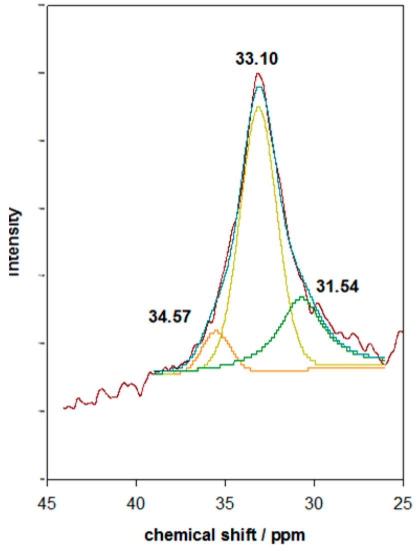

C16ClPG-2.40

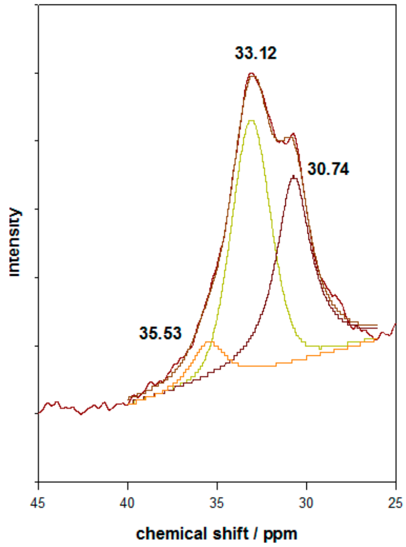

C16OHPG-2.40

Figure S2: Deconvolution of ^{13}C CP MAS peaks in the range of 25 ppm to 45 ppm for C16TMAbr salt and the OC materials.

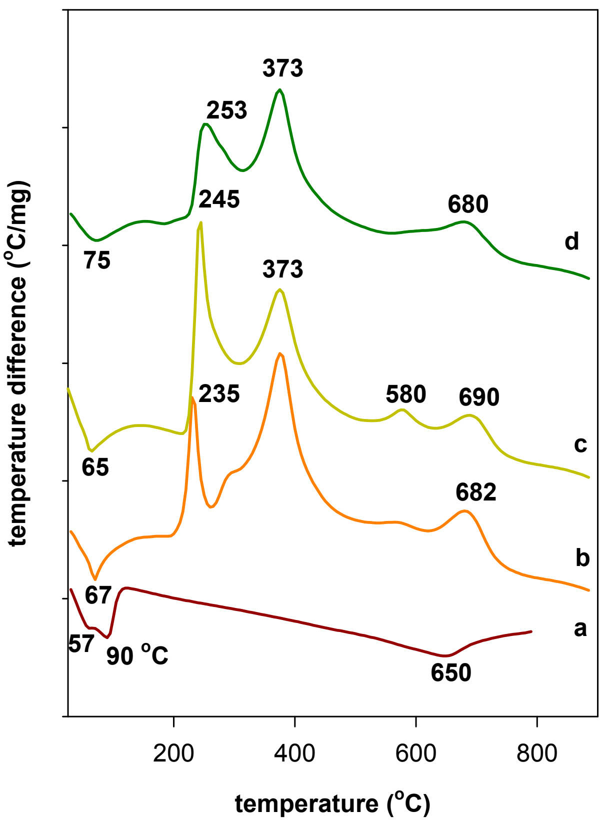

Figure S3. DTA curves of (a) PG clay and organo-clays prepared from different solutions (b) C16TMABr, (c) C16TMACl, and (d) C16TMAOH solutions.

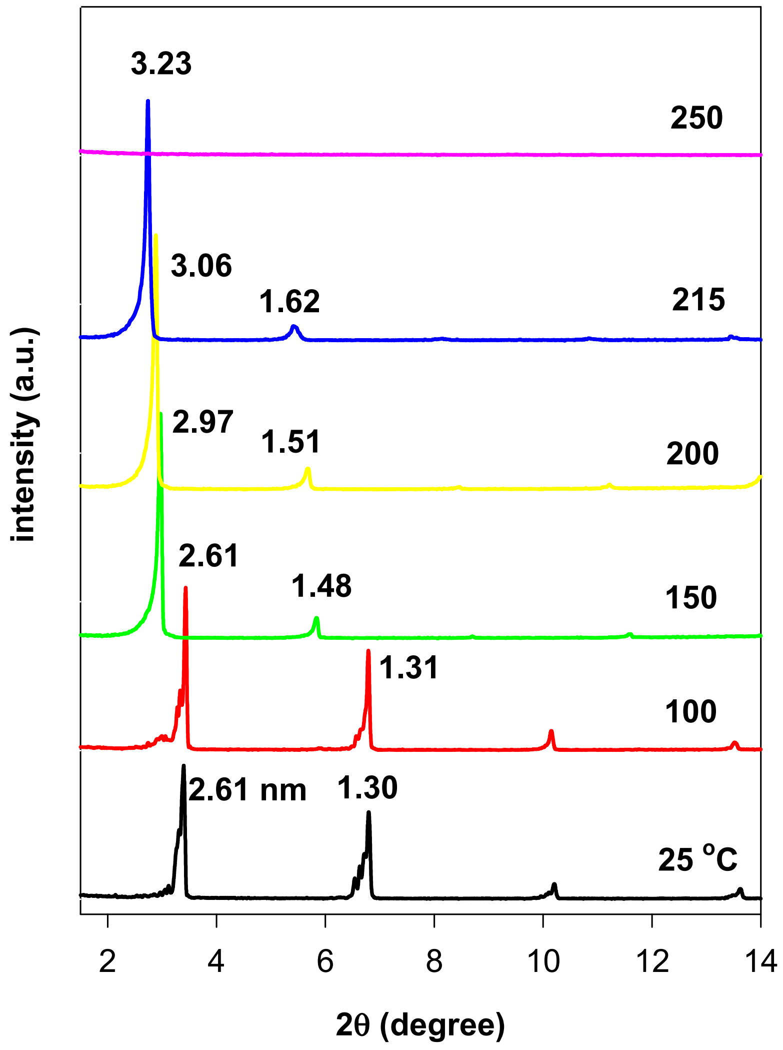

Figure S4. PXRD patterns of C16TMABr salt preheated at different temperatures (°C)

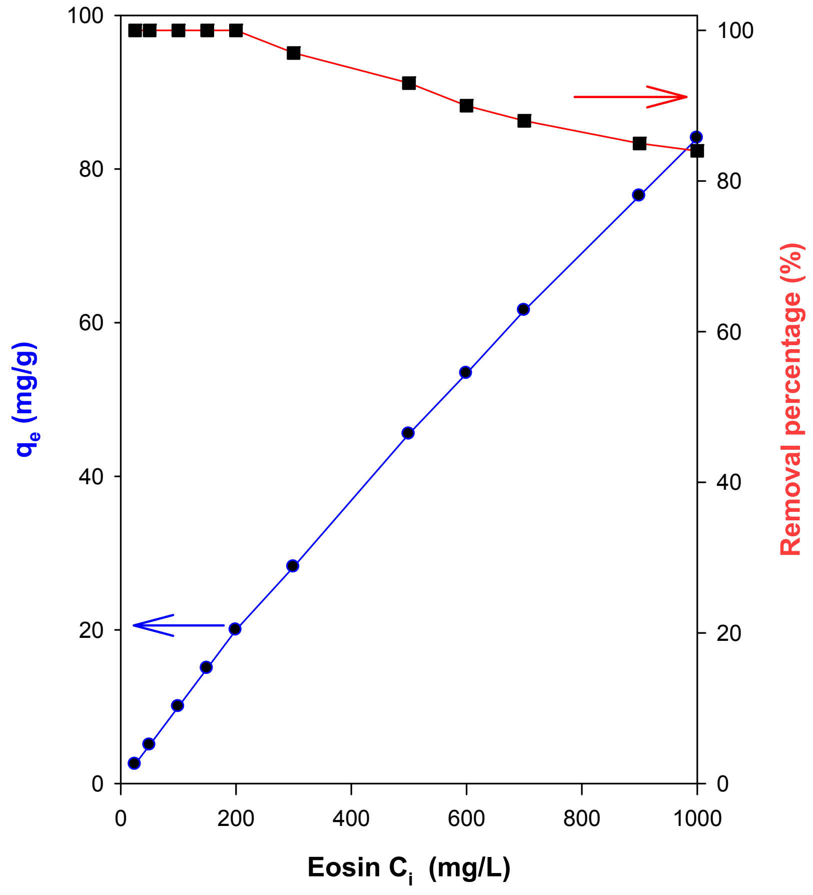

Figure S5. Evolution of removed amount (mg/g) and removal percentage (%) using C16BrPG-2.40 organo-clay.

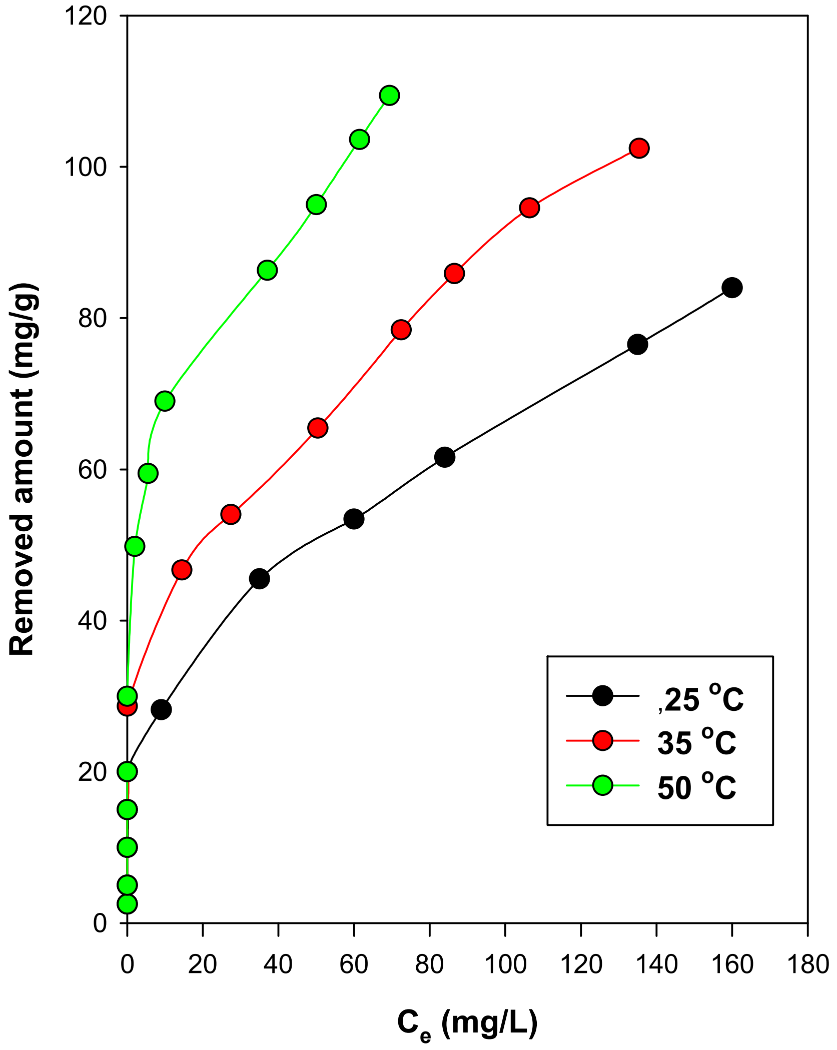

Figure S6. Eosin removal properties of C16BrPG-2.40 organo-clay performed at different temperatures

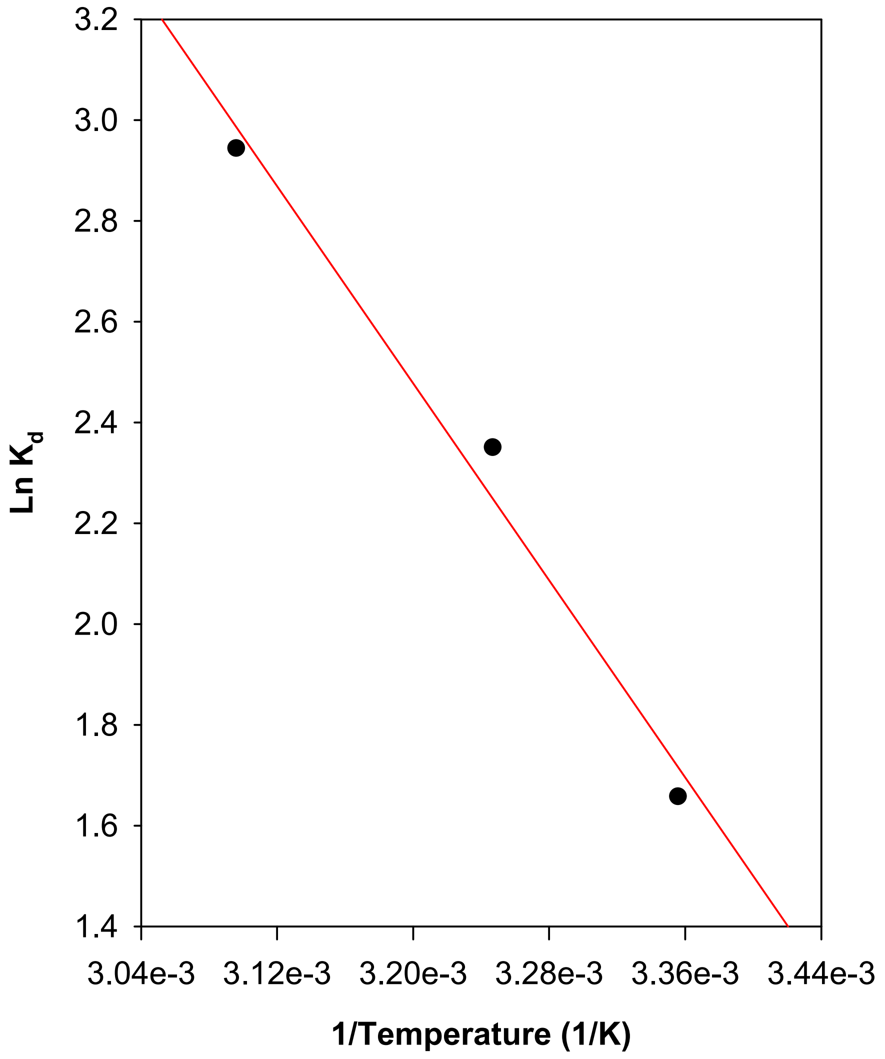

Figure S7. The Van't Hoff plot of eosin removal by C16BrPG-2.40 organo-clay at different temperatures

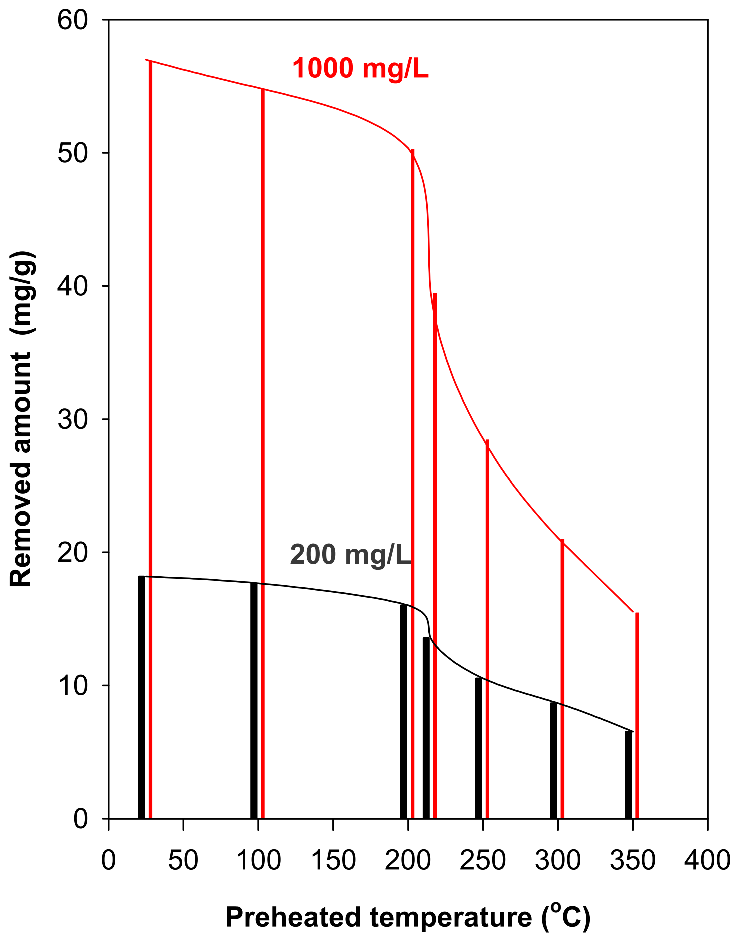

Figure S8. Removal capacity of C16OHPG-2.40 preheated at different temperatures and using two C_i values 200 mg/L (black) and 1000 mg/L (red).
